# Breastfeeding practices and social norms in Kinshasa, Democratic Republic of the Congo: A qualitative study

**DOI:** 10.1371/journal.pgph.0000957

**Published:** 2024-04-16

**Authors:** Pélagie Babakazo, Lina M. Piripiri, Jean-Marie Mukiese, Nelly Lobota, Éric Mafuta

**Affiliations:** 1 Kinshasa School of Public Health, University of Kinshasa, Kinshasa, Democratic Republic of the Congo; 2 Hôpital Général de Référence de Makala, Kinshasa, Democratic Republic of the Congo; PLOS: Public Library of Science, UNITED STATES

## Abstract

**Introduction:**

Breastfeeding has many benefits for both mothers and children. The World Health Organization recommends exclusive breastfeeding for the first six months of life. However, in the Democratic Republic of the Congo, slightly under half of children under six months are exclusively breastfed. This study aimed to describe breastfeeding practices and to explore perceived social norms regarding breastfeeding among mothers in Kinshasa.

**Materials and methods:**

A qualitative descriptive study was conducted in Kinshasa from June to July 2013. This study purposively sampled 54 mothers of infants aged 6 to 12 months, who participated in six focus group discussions. Based on the Theory of Planned Behaviour, the discussion guide explored infant feeding in the first six months, knowledge of breastfeeding, perception of the feasibility of exclusive breastfeeding, and perception of the social norms with regard to exclusive breastfeeding. The content analysis approach was used to analyse data.

**Results:**

Mothers had good breastfeeding knowledge; however, few of them had practised exclusive breastfeeding as recommended during the first six months. Exclusive breastfeeding was considered unfeasible in their context. Barriers to exclusive breastfeeding were reported as baby’s cries, social pressure, warm climate, and poor maternal diet. Social norms were supportive of breastfeeding but unfavourable to exclusive breastfeeding.

**Conclusion:**

In Kinshasa, mothers have a good knowledge of breastfeeding. However, few practise exclusive breastfeeding. Social pressure plays an important role in the cessation of exclusive breastfeeding before six months. In order to improve the practice of exclusive breastfeeding in this context, social and behaviour change programmes should target the entire population rather than mothers only.

## 1. Introduction

Breastfeeding has many advantages for both mothers and children, especially in low and middle-income settings. As reported in the 2016 Lancet series on breastfeeding, some 823 000 child deaths and 20 000 maternal deaths could have been prevented each year by scaling up breastfeeding to a near-universal level [1]. Growing evidence shows that breastfeeding protects against child morbidity and mortality due to infections, increases intelligence and prevents obesity and diabetes [2, 3]. Breastfeeding benefits mothers by improving birth spacing and protecting against breast cancer, type 2 diabetes, and ovarian cancer [2]. Women who breastfed for more than thirty-one months could reduce the risk of ovarian cancer by up to 91.1%, compared to women who breastfeed for less than ten months [4]. In addition, not breastfeeding is associated with an economic loss of around $302 billion per year worldwide [1].

Based on this strong evidence, the World Health Organization (WHO) recommends initiating breastfeeding within one hour of birth, exclusive breastfeeding (EBF) for the first six months of life, and continued breastfeeding for up to two years or beyond, along with nutritionally adequate and safe complementary foods [5]. Exclusive breastfeeding is defined as the practice of giving an infant only breast milk without other liquids (including water) or solids [6]. In the first six months, human milk provides all the energy, nutrients and fluids an infant needs for optimal growth and development [5]. However, despite this recommendation, less than five out of ten infants under six months were exclusively breastfed worldwide between 2014 and 2020 [7]. Even in central African countries, where breastfeeding is common, less than a quarter of infants under six months are exclusively breastfed [8].

Breastfeeding practices are affected by a large variety of factors. The most reported barriers to EBF in low- and middle-income countries are delivery outside a health facility, pre-lacteal feeding, mother’s employment, perceived inadequate maternal nutrition, breastfeeding problems, perceptions of insufficient breast milk production, and lack of support from healthcare providers and relatives, especially fathers [9].

Furthermore, a woman’s decision to breastfeed is a function of her intention to breastfeed and her breastfeeding self-efficacy [10]. Intention to breastfeed, in turn, is influenced by both the woman’s own attitude toward breastfeeding and by her perceptions of what other people think [11]. These socio-cultural beliefs are referred to as "social norms" and are implied group rules about what constitutes appropriate behaviour [12]. Social norms can also be defined as perceived social pressure to perform or not to perform a behaviour [13]. In almost all cultures, breastfeeding is portrayed as the best for babies. However, women often face negative reactions when they breastfeed in public or wish to breastfeed exclusively during the first six months [9, 14].

In the Democratic Republic of the Congo (DRC), the Ministry of Health has signed up to the global strategy for infant and young child feeding and recommends exclusive breastfeeding for six months. However, although almost all children were breastfed (99%) in the 2017–2018 Multiple Indicator Cluster Survey, only 47% were breastfed within one hour of birth, and only 53% of infants under six months were exclusively breastfed [15]. Previous studies in Kinshasa, Sud-Kivu and Kwango identified individual barriers to EBF, such as low level of breastfeeding knowledge, lack of confidence in breastfeeding ability, breastfeeding problems during the first week and the perception that the baby is not getting sufficient milk [16–19].

However, breastfeeding is a social behaviour [20], and therefore strongly affected by social norms [21]. Social norms are “beliefs about which behaviours are appropriate within a given group” [22]. Our behaviours are conditional upon expectations about what others do (empirical expectations/descriptive norms) and expectations about what others think or should do (normative expectations/injunctive norms) [22]. Social norms are driven by the beliefs and/or practices of other people belonging to one’s community. A mother is more likely to breastfeed exclusively for the first six months if she thinks that most mothers in her community exclusively breastfeed their children and/or if she believes that exclusive breastfeeding is approved in her community [23]. Some socio-cultural beliefs make the practice of EBF difficult, as breastfeeding mothers often feel pressured to comply with acceptable practices in their culture [24]. Little is known about social norms related to breastfeeding practices in the DRC. Wood et al. [25] found that in Kinshasa, community norms did not support EBF. A better understanding of these social norms is needed to plan targeted interventions to promote optimal breastfeeding practices, including EBF, in the first six months.

This study was conducted to answer the following central research question: What are breastfeeding practices during the first six months in Kinshasa? The sub-questions were: Do these practices differ according to the socio-economic level of the setting? What are mothers’ perceptions of the feasibility of EBF? What are the social norms regarding breastfeeding practices in Kinshasa? Therefore, this study aimed to describe breastfeeding practices and to explore perceived social norms regarding breastfeeding among mothers in Kinshasa.

## 2. Materials and methods

### 2.1. Study design and sampling

A qualitative descriptive study was conducted in Kinshasa from June to July 2013. Focus group discussions with mothers were used to collect data, as this technique aims to draw from the personal experiences, beliefs, perceptions and attitudes of the participants and thus permit an in-depth understanding of social issues related to the phenomenon under study [26].

In order to be eligible for sampling, the mother had to be at least 18 years old with an infant aged between six and 12 months. As we wanted to collect information about breastfeeding practices during the first six months, we recruited women with a child at least six months old. Furthermore, the child had to be no older than 12 months to minimise recall bias.

Participants were purposively selected using a multi-stage sampling strategy to obtain a diverse range of participants. Kinshasa is subdivided into nine strata based on the chronology of creation and the socio-economic conditions of the towns. In order to have diverse income levels, three health zones with different socio-economic levels were chosen based on this subdivision: the first health zone was in a relatively high-income setting (Lemba), the second in a middle-income setting (Ndjili), and the third in a low-income setting (Kisenso). In each selected health zone, one healthcare facility with a child growth monitoring service was selected to host the focus groups. This selection was based on the attendance rates; the healthcare facility with the highest rate was selected to expect reaching the targeted number of eligible mothers. In each selected healthcare facility, mothers were recruited using a purposive sampling strategy based on their age as well as the age of the child. At least two focus groups had to be held in each healthcare facility in order to reach saturation. Data collection was interrupted after two focus groups had been held in each healthcare facility, as new information was no longer emerging. Each focus group consisted of 6 to 12 mothers.

### 2.2. Recruitment

Prior to holding the focus groups, the research assistant met with the manager of each of the selected healthcare facilities to arrange for the focus groups to be held on a day when the healthcare facility was organising a growth monitoring visit for children under five years. On the appointed day, the nurse in charge of the growth monitoring session informed mothers about the study and directed them to the research team at the end of the visit. The focus groups took place on the same day in the healthcare facility, within thirty minutes of the end of the growth monitoring session.

### 2.3. Conceptual framework

In this study, a conceptual framework was developed to guide data collection and analysis [27]. This conceptual framework was inspired by the Theory of Planned Behaviour, an effective theoretical framework for predicting behavioural change that has also been used to predict breastfeeding behaviour [11, 28]. This conceptual framework highlighted three factors that influence the practice of EBF: the mother’s knowledge of breastfeeding, perception of the feasibility of EBF, and perceived social norms about EBF. These concepts were defined as follows:

**Practice of EBF** refers to the mother who had given her infant only breast milk during the first six months, without any other liquids or solids, except oral rehydration solution or drops/syrups of vitamins, minerals or medicines [6]. To do so, discussions with mothers focused on their children’s nutrition history during the first six months.

**Knowledge of breastfeeding** refers to the mother’s knowledge of the benefits of breastfeeding and her awareness of the WHO recommendation related to EBF.

**Perception of the feasibility of EBF** refers to what the mother thought about her ability to meet the WHO’s recommendation relating to EBF for the first six months. The mother’s perception is referred to as "perceived behavioural control" [29].

**Perceived social norms about EBF** refers to what the mother perceived as collective normative beliefs regarding EBF [11].

### 2.4. Data collection

Focus group and face-to-face interviews were conducted to collect data. The data collection team comprised two female researchers fluent in French and Lingala, the main language spoken in Kinshasa. The facilitator was a medical doctor with a doctorate in public health. Field notes were taken by the research assistant, a nurse experienced in qualitative data collection.

Before starting the focus group discussion, the research assistant conducted face-to-face interviews with the participants to obtain informed consent and to collect socio-demographic characteristics: age, parity, level of education and main occupation. She discussed with each of them what a focus group was, what topic would be discussed, who the other participants were, and how long the focus group session would last. In order to maintain the confidentiality of the research data, which could not be guaranteed due to the nature of a focus group, participants were asked not to repeat what was said in the focus group outside of the meeting. In addition, to maintain anonymity, no real names or other direct identifying information were reported. Participants in the focus group discussions were identified using numbers.

In order to optimise privacy, the focus groups were held in a quiet place away from both healthcare providers and other people. They were conducted in Lingala and audio-recorded with the consent of the participants. The focus group sessions lasted about an hour and a half on average. The questions were open-ended to encourage discussion and to allow the moderator some flexibility to explore specific issues raised. The same discussion questions were used for all focus groups throughout the study.

A debriefing session among the research team was held after each focus group discussion, during which themes, impressions of the findings and procedures were discussed and documented in field notes, and reports were written up. Towards the end of the study, there no new information was emerging.

### 2.5. Data analysis

Socio-demographic data were edited and analysed using Microsoft Excel software. The mothers’ age was summarised using the median and the interquartile range (IQR), while proportions were calculated for categorical data.

Audio recordings were transcribed verbatim in Lingala by the research assistant, checked with the facilitator, supplemented using field notes, and translated into French and finally into English. In order to ensure that the translation was accurate, transcripts were back-translated from English to Lingala by an independent person who was fluent in both languages, and corrections were made where necessary.

The data were analysed using qualitative content analysis by the first and the last authors. The approach was deductive and inductive. Firstly, the deductive approach was used, as we began by identifying key concepts or variables as initial coding categories of analysis using an existing theory [30]. Secondly, for each initial category, the inductive approach was used to identify additional codes through the comments of the participants. For the analyses, we first familiarised ourselves with the data by reading and rereading the transcripts. Then the transcripts were uploaded into Atlas.ti software, version 7.1.4 (Berlin, Germany) for coding, using a codebook developed prior to the start of the focus groups, guided by the categories and subcategories formulated in the data collection tool. Finally, in the query report generated from Atlas.ti, similar responses to each interview question were highlighted with the same colour and therefore grouped together to form common themes. The frequency of similar words or phrases was noted to assist with identifying important themes that helped in understanding the patterns in data. Furthermore, to determine the influence of economic status on breastfeeding practices, results were compared between the three study settings.

### 2.6. Ethical considerations

The study protocol was approved by the Ethical Committee of the Kinshasa School of Public Health (ESP/CE/001/2012). All research procedures in this qualitative descriptive study were in accordance with the Declaration of Helsinki. All participants were fully informed about the nature, the implications of the study and the freedom to opt out at any time should they feel uncomfortable. Moreover, participants voluntarily provided verbal consent to participate and agreed for the discussion to be audio-recorded.

## 3. Results

As mentioned above, two focus groups were organised in each healthcare facility, with six focus groups for the three healthcare facilities. There were 92 mothers approached, and 59 were eligible. Among them, five declined to participate because they had other activities planned. Thus, 54 mothers from the three health zones participated in the study: 22 in Ndjili (41%), 18 in Kisenso (33%) and 14 in Lemba (26%).

### 3.1. Demographic characteristics of the participants

Participant ages ranged from 18 to 43 years, with a median age of 30 years (IQR: 12). Almost two-fifths (39%) of the participants were primiparous. Most (78%) had reached secondary education, and around two-fifths (43%) were housewives (Table 1).

**Table 1:** Demographic characteristics of study participants.

Variables	Kisenso (N = 18)	Ndjili (N = 22)	Lemba (N = 14)	Total (N = 54)
**Age (years),** median (IQR)	27.5 (8.0)	35.5 (14)	29(10)	30.0 (12.0)
**Parity**				
1	05	10	06	21(38.9)
2–3	08	04	05	17(31.5)
≥4	05	08	03	16(29.6)
**Level of education**				
Primary	02	00	00	02(03.7)
Secondary	13	19	10	42(77.8)
University	03	03	04	10(18.5)
**Main occupation**				
Housewife	07	10	06	23(42.6)
Traders	05	07	01	13(24.1)
Aesthetician/Dressmaker	05	02	03	10(18.5)
Student	01	01	02	04(07.4)
Salaried Employee	00	02	02	04(07.4)

### 3.2. Knowledge of breastfeeding

Knowledge of breastfeeding included mothers’ knowledge of the benefits of breastfeeding and awareness of the recommendation relating to EBF. Mothers were also asked about the possible disadvantages of breastfeeding.

#### Benefits of breastfeeding and awareness of the EBF recommendation

All participants recognised that breastfeeding had benefits for the baby. They said that breast milk ensures the child’s good health, growth and intellectual development, protects against infections and meets nutritional needs. In addition, almost all participants were aware of the recommendation that infants should be exclusively breastfed for the first six months. Almost all participants were informed of this recommendation by healthcare providers, while a minority of participants were informed by people in their neighbourhood or by listening to the radio or the television.

#### Disadvantages of breastfeeding

Although most participants felt that breastfeeding had no disadvantages, a few mothers felt that breast milk could "spoil" and cause diarrhoea in the child. For most of them, this could happen if the mother ate too salty or sour foods or if the baby burped on the breast during breastfeeding. For a minority of participants, breast milk could spoil if too much time elapsed between breastfeeds, or the mother became pregnant or had extramarital sexual relationships. A 39-year-old first-time mother said that if a woman had extramarital sex, her child would get *sanga* (FG 5, Ndjili), a disease that occurs in young children and mainly manifests through diarrhoea.

### 3.3. Breastfeeding practices in the first six months

Almost all mothers had initiated breastfeeding, but only a few mothers reported exclusively breastfeeding their babies for the first six months. Exclusive breastfeeding was practised more by participants living in Kinsenso, less in Ndjili and not at all in Lemba.

#### Breastfeeding practices directly after birth

Most participants reported giving "the first yellow-coloured milk" to their babies, although a minority was first afraid to do so because of its yellowish colour. They said to have done so because the colostrum contains vitamins and proteins, protects against infections, strengthens the child, ensures cerebral development, helps the "white milk" to flow in and allows the elimination of meconium. Regarding protection against infection, a 40-year-old first-time mother said, “It’s like the baby’s first vaccine” (FG 6, Ndjili). However, a minority expressed the colostrum before feeding the baby, claiming this milk was "dirty".

#### Administration of foods other than breast milk

The foods other than breast milk given to babies before six months mentioned in the discussions included water, formula milk and porridge.

*Water*. Most participants reported giving water to their children before the age of six months. This practice was more common in Lemba (14/14), followed by Ndjili (14/22) and, finally, Kisenso (9/18). The main reasons mentioned by mothers for doing so were the following–warm climate, people’s talk, insufficient breast milk production and crying babies. A 38-year-old fourth-time mother said, “I wanted to wait until six months before giving water to my baby. However, when he was five months old, his father and paternal aunts asked me to give him water because it was warm” (FG6, Ndjili).

In addition, a minority gave the child water before the age of six months because breast milk is hot and makes the child thirsty after consumption.

*Formula milk and porridge*. Some participants bottle-fed their babies before the age of six months, while most mothers fed them with porridge. Again, bottle-feeding was more common in Lemba (10/14), followed by Ndjili (8/22) and rare in Kisenso (1/18). The same trend was noted for porridge feeding (Lemba:10/14, Ndjili:14/22 and Kisenso:5/18).

Most mothers who had bottle-fed their babies or fed them porridge did so because they felt their milk was insufficient to meet their baby’s needs. They expressed it in these words: “The baby was not growing well”, “he was not gaining weight”, “he was not satisfied with breast milk”, “he used to cry a lot” and “I did not have enough milk”. The mothers thought that their children were not satisfied with breast milk and wanted to eat something else because of certain signs, such as the baby’s gaze following the hand of his mother when she drinks or eats something, and the baby’s belly not increasing in volume. A 26-year-old second-time mother said, “When my baby was three months old, breast milk was not flowing well, her belly was not increasing in size after a feed. So, my mother-in-law asked me to give her porridge” (FG 5, Ndjili).

A minority of mothers who bottle-fed their babies did so to get them used to consuming other foods because they had to return to work.

### 3.4. Perception of the feasibility of the EBF recommendation

#### Mothers’ perception of the EBF recommendation and its feasibility

Almost all participants thought the EBF recommendation was good and commendable because breast milk contains everything the baby needs and the breast milk protects against disease. Regarding its feasibility, most mothers in Kisenso and Ndjili thought that the recommendation was feasible because many mothers managed to comply with it. “It is possible to follow this recommendation because I am the ‘mistress’ (schoolmistress) of the baby; I am the one who decides what is good for him”, said a 24-year-old first-time mother (FG 1, Kisenso). Another participant, a 26-year-old fifth-time mother stated, “I am the mother of the baby. Even though people in the neighbourhood were saying, "Why are you starving your baby?", but I had decided that he would not eat until he was 6 months old. And I did so” (FG 2, Kisenso).

By contrast, in Lemba, most mothers said that EBF for six months was very difficult, if not impossible, because of the baby’s cries and the warm climate. “For me, not giving a child water until the age of six months is like "punishing" him. I’m not convinced that it’s good because it’s too hot. I can accept that the child isn’t fed [food], but he must be given water, he can’t be deprived of water”, said a 23-year-old first-time mother (FG 4, Lemba).

#### Barriers to the practice of EBF in the first six months

For most participants, mothers often failed to meet the recommended duration of EBF because of their babies crying. According to the participants, this crying indicates the baby was not getting sufficient breast milk. The second reason, mentioned by a minority of mothers, was "people’s talk". They say that it is not good for a child to be fed only with breast milk, he or she should also be fed with other foods and liquids. A 23-year-old second-time mother spoke of this social pressure faced by mothers in this way, “The mother may be able to wait up to six months before feeding her baby, but people talk a lot” (FG 3, Lemba).

Other barriers to EBF, mentioned by a minority of participants, were the warm climate and the fact that the mother did not have enough to eat due to the economic crisis. A 24-year-old first-time mother said, “In Kinshasa, the mothers themselves do not eat well. Therefore, the baby will not get full by taking only breast milk. So, it is better for the child to eat for himself. The mother will give him other foods” (FG 5, Ndjili).

### 3.5. Perceived social norms regarding breastfeeding and EBF

#### Breastfeeding in public

Almost all participants stated that if their babies expressed a demand for breast milk in a public space, such as on a bus, they would breastfeed without concerns. A 35-year-old fourth-time mother stated, “I will breastfeed; the child is mine. On the bus, no one will see that it is bad unless you are ashamed to bring out your breast” (FG 3, Lemba).

In contrast, very few participants said they would breastfeed but would cover their breasts for secrecy or protection from evil spirits. In addition, a minority of mothers stated that they would not breastfeed in public mainly out of fear of evil spirits. Referring to this fear, a 36-year-old second-time mother stated that she could not breastfeed in public or expose her breasts because she did not know the "state of soul" of the people around her (FG 2, Kisenso).

#### Perceived social norms towards breastfeeding in public

According to all the focus group participants, people in Kinshasa accept that mothers breastfeed in public. Moreover, the participants asserted that if a baby demands the breast while the mother is on a bus, the other passengers will urge her to breastfeed her baby with statements such as, “Did you steal this baby?” (FG 6, 23-years-old second-time mother) and “Give him/her the breast, it belongs to him/her, why are you hiding it?” (FG 3, 38-years-old, first-time mother).

#### Perceived social norms towards EBF

For almost all participants in group discussions, people in Kinshasa found it unacceptable for children to be exclusively breastfed during the first six months. They are opposed to this practice. Mothers who try to breastfeed exclusively feel pressured to stop doing so. For most participants, if a mother tries to practise EBF, people will tell her that she is "hurting" her baby in vain. They will say, “You are killing your child”, stated a 37-year-old first-time mother (FG 5, Ndlili). Participants also asserted that their mothers tell them, “When we raised you, we gave water and other foods. What bad had happened to you?” (FG 3, 31-year-old third-time mother). However, a minority of participants said that a few of their relatives agreed with the EBF recommendation and encouraged the mothers to follow it.

## 4. Discussion

This study aimed to describe breastfeeding practices and to explore perceived social norms regarding breastfeeding among mothers in Kinshasa. The results show that although mothers had adequate knowledge of breastfeeding, few reported exclusively breastfeeding their babies for the first six months. Mothers perceived that people in Kinshasa supported breastfeeding. However, they found it unacceptable to exclusively breastfeed children for the first six months.

### 4.1. Knowledge of breastfeeding

Participants in this study had good knowledge of breastfeeding benefits for the child. They knew that children had to be exclusively breastfed until the age of six months. Thus, this study builds on previous studies that reported good breastfeeding knowledge among mothers in sub-Saharan African countries [31–33]. The main source of information related to breastfeeding in this study was healthcare providers. Similarly, findings from previous studies in sub-Saharan Africa highlighted that healthcare providers are the primary source of information on optimal breastfeeding practices [34, 35]. The adequate knowledge of optimal breastfeeding reported in this study suggests that infant feeding education was successfully provided to mothers during antenatal and postnatal care visits.

However, although the participants knew the benefits of breastfeeding for the child, none mentioned the benefits for the mother. A similar result has been previously reported in Ghana [31]. In addition, participants in this study reported that breast milk can "spoil" and therefore become unsafe for the baby. This can especially happen if the mother has extramarital sexual relationships or if the baby burps on the breast. This cultural belief was also reported in Kenya [36] and Tanzania [37]. Breastfeeding counselling provided by healthcare providers should seek to address this knowledge gap and to correct misconceptions about breast milk.

### 4.2. Breastfeeding practices

In this study, almost all babies had been breastfed; however, few were exclusively breastfed during the first six months. This result is similar to those reported in previous studies in DRC [16–19]. This gap between breastfeeding knowledge and practices has also been reported in Kenya [38], Tanzania [39] and Ghana [31]. Future research should focus on this gap, which is probably due to cultural barriers. Thus, specific interventions could be carried out to address it.

During this study, mothers in a low-income setting reported higher rates of EBF than those in a relatively high-income setting. Similar results were reported in other countries in sub-Saharan Africa [40–42].

One could speculate that mothers with low socio-economic status may be unable to afford breast milk substitutes and other foods and liquids. Thus, EBF could be the only option available to them. In order to improve the practice of EBF in Kinshasa, special attention must be paid to these mothers with a high socio-economic level.

#### Reasons for discontinuing EBF before six months

Findings from this study showed that mothers gave water to their children because the mothers thought the baby was thirsty due to the warm climate. In warm climates, mothers do not abstain from giving water to their babies because of the heat. This misconception has been previously reported in Kinshasa [25], and Ghana [43]. However, during the first six months of life, even in warm climates, healthy infants who are exclusively breastfed do not need water [44]. Moreover, in Kinshasa, access to safe drinking water is limited. Therefore, giving water to infants is a risk factor for gastro-intestinal infections. Mothers should be made aware of this evidence and encouraged not to give their infants water for the first six months unless medically indicated.

Formula milk and porridge were given to the infants before six months because the mother felt that her breast milk was insufficient to meet the baby’s needs. The perception of insufficient breast milk supply is one of the most commonly reported reasons for the early introduction of foods other than breast milk in the DRC [17] and in other sub-Saharan African countries [24]. However, this perception of insufficient milk supply is more perceived than real [45], generally due to low maternal breastfeeding self-efficacy[46, 47]. Moreover, this perception can also be explained by a misinterpretation of certain signs considered by mothers in Kinshasa as proof that the child wants to eat something else. All these misinterpretations should be taken into account during education sessions, and mothers should be reassured about their ability to breastfeed.

Some mothers in this study bottle-fed their babies because they had to resume work. Resuming school or work is the second most commonly reported reason for the early introduction of other foods and liquids [9, 24, 48]. Employed mothers face the challenge of managing breastfeeding and work. In Kinshasa, this challenge is particularly greater due to the absence of legislation in favour of breastfeeding and the absence of baby-friendly spaces in the workplace. In this context, the only option to improve the practice of exclusive breastfeeding among employed women would be to encourage them to express breast milk at home before leaving for work, safely store the expressed breast milk and then cup-feed the baby. However, the most appropriate intervention would be to extend paid maternity leave to the duration recommended for EBF, and to make the workplace breastfeeding friendly.

### 4.3. Perception of the feasibility of the EBF recommendation

For participants in this study, the EBF recommendation is beneficial but not feasible. A similar result was reported in a Tanzanian study [49]. The mothers were hostile to this recommendation mainly because it deprives children of water, and they believe "water is life". No one can live without water, and a baby cannot survive for the first six months without drinking water. Similar beliefs have been reported in other countries in sub-Saharan Africa, including the DRC [25, 38, 43]. This misconception can be explained by the mothers’ limited knowledge about the water composition of breast milk. Therefore, breastfeeding counselling should address this knowledge gap by using evidence to reassure mothers that breast milk alone is sufficient to meet the baby’s water needs for the first six months.

#### Barriers to the practice of EBF in the first six months

This study showed that mothers in Kinshasa often failed to meet the recommended duration of EBF because of the baby’s cries, perceived to be a sign of dissatisfaction with breast milk. The second most common barrier to the practice of EBF mentioned in this study was "people’s talk". Through their statements, people often put pressure on the mother and discourage her from practising EBF. This social pressure is even greater in Kinshasa, where the larger family system is important, especially in the outlying districts. Extended family members and even neighbours greatly influence the choice and adoption of different behaviours, including the practice of EBF. Thus, the mother would start giving water and other foods to her baby to comply with what her social environment asks her to do. Similar results were reported in DRC and South Africa [25, 48]. This social pressure can also explain the gap between breastfeeding knowledge and practices noted in this study. Therefore, extending social and behaviour change communication on infant feeding to the entire population may be a promising strategy to improve the practice of EBF in Kinshasa, especially since mothers in this study reported that a minority of their relatives, who were aware of the recommendation on the duration of EBF, encouraged them to follow it.

Other barriers to the recommended duration of EBF mentioned by participants in this study were the warm climate and the poor maternal diet due to the economic crisis. Similarly, poor maternal diet has been reported previously as a barrier to EBF in the DRC [17] and other countries in sub-Saharan Africa [24, 39, 49]. Mothers believe that the quality and quantity of breast milk may deteriorate if the mother does not eat properly, and the child must therefore be given additional foods. This misconception should also be addressed through social and behaviour change communication, as breast milk has a fairly consistent composition and is almost unaffected by the mother’s diet [50]. Breastfeeding mothers are advised to eat well, but this is mostly for their energy balance [51].

### 4.4. Perceived social norms towards EBF and breastfeeding in public

The results of this study showed that breastfeeding in public was considered acceptable in Kinshasa. Moreover, the majority of mothers were comfortable breastfeeding in public. However, some could do so while covering their breasts to protect themselves from evil spirits and avoid discomforting others. Similar findings have been previously reported [37, 52]. This discomfort of breastfeeding in public is due to the sexualisation of the female breast. Breasts are considered a private part of the body which should not be made visible in public [53].

In contrast, people in Kinshasa did not accept that children be exclusively breastfed for the first six months. They consider the practice of EBF to be "child abuse", and they usually affirm it with words that mothers experience as "social pressure". Wood et al. [25] also found that community norms in Kinshasa did not support EBF. Furthermore, participants in this study pointed out that older generation mothers preferred that the current mothers adopt the same infant feeding practices that they had. The grandmothers did not practise EBF and their children grew up and were healthy. A similar result was reported in a Ghanaian study [43]. This result confirms previous evidence that grandmothers have the ability to influence the practice of EBF [54]. These results again suggest that intervention to promote optimal breastfeeding practices should target the entire community in general and grandmothers in particular.

### 4.5. Strengths and limitations

The results of this study should be interpreted in the context of certain limitations. First, the data used were collected in 2013 and may appear outdated. However, we believe that the results of this study are still valuable today, as the national rate of mothers meeting the global recommendation for exclusive breastfeeding has been approximately 50% from 2013 to 2018 [15, 55].

Second, social desirability bias may have occurred in this study because mothers were not interviewed individually, and focus groups were conducted in health facilities. Thus, some mothers might have avoided discussing aspects of their breastfeeding practices they considered negative. In order to minimize this bias, the discussion groups were moderated by independent persons away from the healthcare providers, and the participants were reminded of their confidentiality. Third, information about infant feeding in the first six months was collected retrospectively, so recall bias could not be completely avoided. Finally, the study excluded other people who influence infant feeding practices, especially fathers and grandmothers.

However, this study included mothers from different ethnic groups and socio-economic settings and is one of the few studies using qualitative methods to understand breastfeeding practices in Kinshasa. Findings from this study can be used to develop social and behaviour change programmes that target local barriers to EBF. Another strength of this study is that a theory was used in the design and analysis.

## 5. Conclusion

In Kinshasa, mothers are knowledgeable of the benefits of breastfeeding and the recommendation relating to EBF. However, this knowledge is not translated into practice. Breastfeeding is common, but EBF for six months is rare. Challenges to meeting the recommended duration of EBF include the warm climate, dissatisfaction with breast milk, social pressure and poor maternal diet. Although breastfeeding, even in public, is sociably acceptable, the social norms find EBF unacceptable and play an important role in its discontinuation before six months. In order to improve practising EBF in this context, social and behaviour change programmes should target the entire population, not just mothers.

## Supporting information

S1 File Analysis matrix. (PDF)

S2 File Moderator guide. (DOCX)
