# Supplementary material for: Breastfeeding practices and social norms in Kinshasa, Democratic Republic of the Congo: A qualitative study
Source: PLOS Glob Public Health. 2024 Apr 16;4(4):e0000957. doi: 10.1371/journal.pgph.0000957 (PMC11020689; doi:10.1371/journal.pgph.0000957)
Supplement: S1 File — (PDF) [file pgph.0000957.s001.pdf]

Breastfeeding practices and social norms in Kinshasa, Democratic Republic of the Congo: A qualitative approach

ANALYSIS MATRIX

1. Breastfeeding Knowledge

1.1. Advantages and disadvantages of breastfeeding

Setting	FG	Advantages of breastfeeding	Disadvantages of breastfeeding
**KISENSO**	**FG1**	Good growth (7/11)<br>Vitamins/proteins (2/11)<br>Brain/Intelligence (2/11)<br>Strengthen/strong (2/11)<br>Contains everything (2/11)	No disadvantages (8/11)<br>Spoiled/ Diarrhoea (3/11)<br>- Salty foods (2/4)<br>- Extramarital sexual relationships (1/4)<br>- Baby burped on the breast (2/4)<br>- Bad foods (1/4)<br>- Pregnancy (1/4)<br>Diarrhoea (4/4)
	**FG2**	Good growth (1/7)<br>Vitamins/proteins (2/7)<br>All nutrients/needs (2/7)<br>Brain/Intelligence (2/7)<br>Protects/Antibody (1/7)	No disadvantages (5/7)<br>Spoiled (2/7)<br>- Salty foods (2/2)<br>- sour foods (1/2)<br>- Baby burped on the breast (2/2)<br>- Bad foods (2/2)
	**Summary for KISENSO**	*Has benefits (19/18)*<br>- *Good growth (8/18)*<br>- *Vitamins/proteins (4/18)*<br>- *Brain/Intelligence (4/18)*<br>- *Contains everything (4/18)*<br>- *Strengthen/strong (2/18)*<br>- *Protects/Antibody (1/18)*	*No disadvantages (13/18)*<br>*Can become harmful (5/18)*<br>- *Spoiled (5/5)*<br>- *Salty foods (4/5)*<br>- *Baby burped on the breast (4/5)*<br>- *Bad foods (3/5)*<br>- *Extramarital sexual relationships (1/5)*<br>- *sour foods (1/5)*<br>- *Pregnancy (1/5)*<br>- *Diarrhoea (4/5)*
**LEMBA**	**FG3**	Good health (1/7)<br>Strengthen/strong (3/7)<br>Good growth (5/7)<br>Brain/Intelligence (2/7)<br>Protects/Antibody (1/7)	No disadvantages (2/6)<br>Diarrhoea (4/6)<br>- Uncleaned breasts (2/5)<br>- Bad foods (2/5)
	**FG4**	Good health (2/7)<br>Strengthen/strong (1/7)<br>Good growth (3/7)<br>Brain/Intelligence (4/7)<br>Protects/Antibody (1/7)<br>Contains everything (1/7)	No disadvantages (4/7)<br>Spoiled (1/7)<br>- Pregnancy (1/1)<br>Diarrhoea (2/7)<br>- Uncleaned breasts (2/2)
	**Summary for LEMBA**	*Has benefits (14/14)*<br>- *Good growth (8/14)*<br>- *Brain/Intelligence (6/14)*<br>- *Strengthen/strong (4/14)*<br>- *Good health (3/14)*<br>- *Protects/Antibody (2/14)*<br>- *Contains everything (1/14)*	*No disadvantages (6/13)*<br>*Can become harmful (7/13)*<br>- *Spoiled (1/78)*<br>- *Pregnancy (1/1)*<br>- *Diarrhoea (6/7)*<br>- *Uncleaned breasts (4/7)*<br>- *Bad foods (2/7)*
**NDJILI**	**FG5**	Good growth (2/11)<br>Good health (2/11)<br>Best/More than (3/11)	No disadvantages/Nothing (8/11)<br>Spoiled/ Diarrhoea/vomica (3/11)<br>- Salty foods (2/3)

Setting	FG	Advantages of breastfeeding	Disadvantages of breastfeeding
		Brain/Intelligence (3/11)<br>Contains everything (1/11)<br>Strengthen/strong (1/11)<br>Vitamins/proteins (4/11)	- sour foods (1/3)<br>- Baby burped on the breast (1/3)<br>- Uncleaned breasts (1/3)<br>- Extramarital sexual relationships (1/3)<br>- Not breast-feed for long time (1/3)
	**FG6**	Vitamins/proteins (1/11)<br>Good growth (3/11)<br>Good health (2/11)<br>Contains everything (1/11)<br>Protects/Antibody (7/11)<br>Strengthen/strong (1/11)	No disadvantages/Nothing (3/6)<br>Spoiled/ Diarrhoea (3/3)<br>- Salty foods (2/3)<br>- Not breast-feed for long time (1/3)<br>- Sun rays (1/3)<br>- Bad foods (1/3)
	**Summary for NDJILI**	*Has benefits (22/22)*<br>- *Protects/Antibody (7/22)*<br>- *Good growth (5/22)*<br>*Vitamins/proteins (5/22)*<br>- *Good health (4/22)*<br>- *Best/More than (3/22)*<br>- *Brain/Intelligence (3/22)*<br>- *Contains everything (2/22)*<br>- *Strengthen/strong (2/22)*	*No disadvantages/Nothing (11/17)*<br>*Can become harmful (6/17)*<br>- *Spoiled (6/6)*<br>- *Salty foods (4/6)*<br>- *Not breast-feed for long time (2/6)*<br>- *sour foods (1/3)*<br>- *Baby burped on the breast (1/3)*<br>- *Uncleaned breasts (1/3)*<br>- *Extramarital sexual relationships (1/3)*<br>- *Sun rays (1/3)*<br>- *Bad foods (1/3)*
	**Summary for all FG**	*Has benefits (54/54)*<br>- *Good growth (21/54)*<br>- *Brain/Intelligence (13/54)*<br>- *Protects/Antibody (10/54)*<br>- *Vitamins/proteins (9/54)*<br>- *Strengthen/strong (8/54)*<br>- *Contains everything (7/54)*<br>- *Good health (7/54)*<br>- *Best/More than (3/54)*	*No disadvantages/Nothing (30/48)*<br>*Can become harmful (18/48)*<br>- *Spoiled (13/18) => Diarrhoea*<br>- *Salty foods (8/18)*<br>- *Not breast-feed for long time (2/18)*<br>- *sour foods (2/18)*<br>- *Baby burped on the breast (5/18)*<br>- *Extramarital sexual relationships (2/18)*<br>- *Sun rays (1/18)*<br>- *Bad foods (6/18)*<br>- *Pregnancy (2/18)*<br>- *Diarrhoea (5/18)*<br>- *Uncleaned breasts (5/5)*

1.2. Awareness of recommended duration of EBF

Setting	FG	Had ever heard about the EBF recommendation? Where?	
**KISENSO**	**FG1**	Yes (11/11)<br>- Heath care facility (11/11)<br>- People/City/neighbour (1/11)<br>- Radio (1/11)<br>- Television (1/11)	
	**FG2**	Yes (7/7)<br>- Heath care facility (7/7)<br>- Television (1/7)	
	**Summary for KISENSO**	*Yes (18/18)*<br>- *Heath care facility (18/18)*<br>- *Television (2/18)*<br>- *People/City/neighbour (1/18)*<br>- *Radio (1/18)*	

Setting	FG	Had ever heard about the EBF recommendation? Where?	
LEMBA	FG3	No (1/6)<br>Yes (5/6)<br>- Heath care facility (4/5)<br>- Television (1/5)	
	FG4	Yes (7/7)<br>- Heath care facility (4/7)<br>- People/City/neighbour (2/7)<br>- Television (1/7)	
	Summary for LEMBA	*No (1/13)*<br>*Yes (12/13)*<br>- *Heath care facility (8/12)*<br>- *Television (2/12)*<br>- *People/City/neighbour (2/12)*	
NDJILI	FG5	Yes (11/11)<br>- Heath care facility (10/11)<br>- People/City/neighbour (3/11)<br>- Radio (3/11)<br>- Television (3/11)<br>- Mother in law (1/11)	
	FG6	Yes (11/11)<br>- Heath care facility (9/11)<br>- People/City/neighbour (1/11)<br>By myself (1/11)	
	Summary for NDJILI	*Yes (22/22)*<br>- *Heath care facility (19/22)*<br>- *People/City/neighbour (4/22)*<br>- *Radio (3/22)*<br>- *Television (3/22)*<br>- *Mother in law (1/22)*<br>*By myself (1/22)*	
	Summary for all FG	*No (1/53)*<br>*Yes (52/53)*<br>- *Heath care facility (46/52)*<br>- *Television (7/52)*<br>- *People/City/neighbour (7/52)*<br>- *Radio (4/52)*<br>- *Mother in law (1/52)*<br>- *By myself (1/52)*	

2. Infant feeding practices during the first six months

2.1. Administration of colostrum

Setting	FG	Administration of colostrum	Importance of colostrum
KISENSO	FG1	Saw/Gave (4/7)<br>Gave the breast/Did not see (2/7)<br>Did not give (1/7)<br>- Expressed and waited the white milk	Important/good (2/7)<br>Vitamins/proteins (2/7)<br>Brain/intelligence (1/7)<br>Don't know (1/7)<br>Not important/armful (1/7)
	FG2	Saw/Gave (6/7)<br>Gave the breast/Did not see (1/7)	Important/good (3/7)<br>Vitamins/proteins (2/7)<br>Strengthen (1/7)<br>Don't know (1/7)

Setting	FG	Administration of colostrum	Importance of colostrum
	**Summary for KISENSO**	*Saw/Gave (10/14)*<br>*Gave the breast/Did not see (3/14)*<br>*Did not give (1/14)*<br>- *Expressed and waited the white milk*	*Beneficial for the bay (11/14)*<br>- *Important/good (5/11)*<br>- *Vitamins/proteins (4/11)*<br>- *Strengthen (1/11)*<br>- *Brain/intelligence (1/11)*<br><br>*Don't know (2/14)*<br>*Not important/armful (1/14)*
**LEMBA**	**FG3**	Saw/Gave (3/7)<br>Gave the breast/Did not see (3/7)<br>Did not give (1/7)<br>- Baby in a baker	Don't know (4/7)<br>Important (3/7)
	**FG4**	Saw/Gave (4/7)<br>Gave the breast/Did not see (3/7)	Important/good (3/7)<br>Vitamins/proteins (2/7)<br>Brain/intelligence (1/7)<br>Helps the white milk to flow (1/7)<br>Eliminates black stool (1/7)<br>Don't know (1/7)
	**Summary for LEMBA**	*Saw/Gave (7/14)*<br>*Gave the breast/Did not see (6/14)*<br>*Did not give (1/14)*<br>- *Baby in a baker*	*Beneficial for the bay (9/14)*<br>- *Important/good (6/9)*<br>- *Vitamins/proteins (2/9)*<br>- *Brain/intelligence (1/9)*<br>- *Helps the white milk to flow (1/9)*<br>- *Eliminates black stool (1/9)*<br><br>*Don't know (5/14)*
**Ndjili**	**FG5**	Saw/Gave (6/11)<br>- Afraid (2/6)<br>Gave the breast/Did not see (5/11)	Don't know (5/11)<br>Important/good (3/11)<br>Vitamins/proteins (2/11)<br>Brain/intelligence/memory (1/11)<br>Strengthen (1/11)
	**FG6**	Saw/Gave (9/11)<br>Gave the breast/Did not see (2/11)	Protects/First vaccine/antibody (6/11)<br>Important/good (5/11)<br>Don't know (2/11)
	**Summary for NDJILI**	*Saw/Gave (15/22)*<br>- *Afraid (2/15)*<br>*Gave the breast/Did not see (7/22)*	*Beneficial for the bay (15/22)*<br>- *Important/good (8/15)*<br>- *Vitamins/proteins (2/15)*<br>- *Brain/intelligence/memory (1/15)*<br>- *Strengthen (1/15)*<br>- *Protects/First vaccine/antibody (6/15)*<br><br>*Don't know (7/22)*
	**Summary for all FG**	*Saw/Gave (32/50)*<br>- *Afraid (2/32)*<br>*Gave the breast/Did not see (16/50)*<br>*Did not give (2/50)*<br>- *Expressed and waited the white milk (1/2)*<br>- *Baby in a baker (1/2)*	*Beneficial for the bay (35/50)*<br>- *Important/good (19/35)*<br>- *Vitamins/proteins (8/35)*<br>- *Protects/First vaccine/antibody (6/35)*<br>- *Strengthen (5/35)*<br>- *Brain/intelligence/memory (2/35)*<br>- *Helps the white milk to flow (1/35)*<br>- *Eliminates black stool (1/35)*<br><br>*Don't know (14/50)*<br>*Not important/armful (1/50)*

2.2. Administration of water and formula milk

Setting	FG	Administration of water	Administration of formula milk
**KISENSO**	**FG1**	At birth (2/11)<br>- Breast milk is hot (1/3)<br>- Grand-mother (1/3)<br>- Baby was crying a lot (1/3)<br>1 month (2/11)<br>- Baby was crying a lot (1/2)<br>- People was talking a lot (1/2)<br>- Grand-mother (1/2)<br>3 months (2/11)<br>- Baby was disturbing (1/2)<br>- Grand-mother (1/2)<br>6 months (5/11)	6 months (5/11)<br>7 months (1/11)<br>8 months (1/11)<br>Never fed with formula milk (4/11)
	**FG2**	At birth (1/7)<br>- Breast-milk was not flowing (1/1)<br>2 months (1/7)<br>- Baby was crying a lot (1/1)<br>4 months (1/7)<br>- Warmth/very hot (1/1)<br>6 months (4/7)	4 months (1/7)<br>- Supplement breast milk (1/1)<br>6 months (6/7)
	**Summary for KISENSO**	*At birth (3/18)*<br>- *Breast milk is hot (1/4)*<br>- *Grand-mother (1/4)*<br>- *Baby was crying a lot (1/4)*<br>- *Breast-milk was not flowing (1/4)*<br>*1 month (2/18)*<br>- *Baby was crying a lot (1/2)*<br>- *People was talking a lot (1/2)*<br>- *Grand-mother (1/2)*<br>*2 months (1/18)*<br>- *Baby was crying a lot (1/1)*<br>*3 months (2/18)*<br>- *Baby was disturbing (1/2)*<br>- *Grand-mother (1/2)*<br>*4 months (1/18)*<br>- *Warmth/very hot (1/1)*<br>*6 months (9/18)*<br><br>***At least 6 months (9/18)***<br>***Before six months (9/18)***<br>- *Breast milk is hot (1/9)*<br>- *Grand-mother (3/9)*<br>- *Baby was crying a lot (3/9)*<br>- *Baby was disturbing (1/9)*<br>- *Breast-milk was not flowing (1/9)*<br>- *People was talking a lot (1/9)*<br>- *Warmth/very hot (1/9)*	*4 months (1/18)*<br>- *Supplement breast milk (1/1)*<br>*6 months (11/18)*<br>*7 months (1/18)*<br>*8 months (1/18)*<br>*Never fed with formula milk (4/18)*<br><br>***At least 6 months (17/18)***<br>***Before six months (1/18)***<br>- *Supplement breast milk (2/1)*
**LEMBA**	**FG3**	At birth (2/7)<br>- My willingness (1/2)<br>- Breast-milk was not flowing (1/2)	1 month (4/7)<br>- Low growth/ weight stationary (2/4)

Setting	FG	Administration of water	Administration of formula milk
		2 months (2/7)<br>- Baby was crying a lot (1/2)<br>- Warmth/very hot (2/2)<br>3 months (2/7)<br>- Recommend by health care providers (1/2)<br>- Baby was crying a lot (1/2)<br>4 months (1/7)<br>- Warmth/very hot (1/1)	- Return to work (2/4)<br>- For the baby to become accustomed (1/4)<br>- People was talking (1/4)<br>- Recommendations from providers (1/4)<br>- Baby was crying a lot (1/4)<br>2 months (1/7)<br>- Return to school (1/1)<br>5 months (1/7)<br>- Baby was crying a lot (1/1)<br>- Baby was not getting full (1/1)<br>Never fed with formula milk (1/7)
	**FG4**	At birth/while in maternity (6/7)<br>- Grand-mother (1/6)<br>- Breast-milk was not flowing (2/6)<br>- Warmth/very hot (2/6)<br>- Others mothers were giving (1/6)<br>2 months (1/7)<br>- Baby was crying a lot (1/1)	1 month (1/7)<br>- Not enough breast-milk (1/1)<br>2 months (2/7)<br>- Return to work (1/2)<br>- For the baby to become accustomed (1/2)<br>- Baby was suckling a lot (1/2)<br>4 months (1/7)<br>- Baby was not getting full (1/1)<br>Never fed with formula milk (3/7)
	**Summary for LEMBA**	*At birth/while in maternity (8/14)*<br>- *Breast-milk was not flowing (3/8)*<br>- *Warmth/very hot (2/6)*<br>- *My willingness (1/8)*<br>- *Grand-mother (1/8)*<br>- *Others mothers were giving (1/6)*<br>*2 months (3/14)*<br>- *Baby was crying a lot (2/3)*<br>- *Warmth/very hot (2/3)*<br>*3 months (2/14)*<br>- *Recommend by health care providers (1/2)*<br>- *Baby was crying a lot (1/2)*<br>*4 months (1/14)*<br>- *Warmth/very hot (1/1)*<br><br>***At least 6 months (0/14)***<br>***Before six months (14/14)***<br>- *Warmth/very hot (5/14)*<br>- *Breast-milk was not flowing (3/14)*<br>- *Baby was crying a lot (3/14)*<br>- *My willingness (1/14)*<br>- *Grand-mother (1/14)*<br>- *Others mothers were giving (1/14)*<br>- *Recommend by health care providers (1/2)*	*1 month (5/14)*<br>- *Low growth/ weight stationary (2/5)*<br>- *Return to work (2/5)*<br>- *For the baby to become accustomed (1/5)*<br>- *People was talking (1/5)*<br>- *Recommendations from providers (1/5)*<br>- *Baby was crying a lot (1/5)*<br>- *Not enough breast-milk (1/5)*<br>*2 months (3/14)*<br>- *Return to school (1/3)*<br>- *Return to work (1/3)*<br>- *For the baby to become accustomed (1/3)*<br>- *Baby was suckling a lot (1/3)*<br>*4 months (1/14)*<br>- *Baby was not getting full (1/1)*<br>*5 months (1/14)*<br>- *Baby was crying a lot (1/1)*<br>- *Baby was not getting full (1/1)*<br>*Never fed with formula milk (4/14)*<br><br>***At least 6 months (4/14)***<br>***Before six months (10/14)***<br>- *Low growth/ weight stationary (2/10)*<br>- *Return to school (1/10)*<br>- *Return to work (3/10)*<br>- *For the baby to become accustomed (2/10)*<br>- *Baby was crying a lot (2/10)*

Setting	FG	Administration of water	Administration of formula milk
			- Baby was not getting full (2/10)<br>*- Baby was suckling a lot (1/10)*<br>*- People was talking (1/10)*<br>*- Recommendations from providers (1/10)*<br>*- Not enough breast-milk (1/10)*
**Ndjili**	**FG5**	At birth (5/11)<br>- Baby was thirsty (1/5)<br>- Breast-milk is hot (2/5)<br>- Breast-milk was not flowing (1/5)<br>- Baby was receiving substitute (1/5)<br>2 months (3/11)<br>- People was talking a lot (2/3)<br>- Warmth/very hot (1/3)<br>6 months (3/11)	3 days (1/11)<br>- Baby was not getting full (1/1)<br>- Not enough breast-milk (1/1)<br>1 month (2/11)<br>- Baby was not getting full (1/1)<br>- Not enough breast-milk (1/1)<br>2 months (1/11)<br>- Baby was not getting full (1/1)<br>6 months (2/11)<br>9 months (1/11)<br>Never fed with formula milk (4/11)
	**FG6**	At birth (2/11)<br>- Breast-milk not flowing (2/2)<br>2 months (1/11)<br>- Warmth/very hot (1/1)<br>3 months (1/11)<br>- Warmth/very hot (1/1)<br>4 months (1/11)<br>- Warmth/very hot (1/1)<br>5 months (1/11)<br>- Feather and hunts (1/1)<br>6 months (5/11)	2 days (1/11)<br>- Not enough breast-milk (1/1)<br>2 months (1/11)<br>- Return to work (1/1)<br>3 months (2/11)<br>- Low growth/ weight stationary (2/2)<br>6 months (7/11)
	**Summary for NDJILI**	*At birth (7/22)*<br>*- Breast-milk was not flowing (3/7)*<br>*- Breast-milk is hot (2/7)*<br>*- Baby was receiving substitute (1/7)*<br>*- Baby was thirsty (1/7)*<br>*2 months (4/22)*<br>*- People was talking a lot (2/4)*<br>*- Warmth/very hot (2/4)*<br>*3 months (1/22)*<br>*- Warmth/very hot (1/1)*<br>*4 months (1/22)*<br>*- Warmth/very hot (1/1)*<br>*5 months (1/22)*<br>*- Father and hunts (1/1)*<br>*6 months (8/22)*<br><br>***At least 6 months (8/22)***<br>***Before six months (14/22)***<br>*- Warmth/very hot (4/14)*<br>*- Breast-milk was not flowing (3/7)*<br>*- Breast-milk is hot (2/7)*<br>*- People was talking a lot (2/4)*<br>*- Father and hunts (1/1)*<br>*- Baby was receiving substitute (1/7)*<br>*- Baby was thirsty (1/7)*	*2 days (1/22)*<br>*- Not enough breast-milk (1/1)*<br>*3 days (1/22)*<br>*- Baby was not getting full (1/1)*<br>*- Not enough breast-milk (1/1)*<br>*1 month (2/11)*<br>*- Baby was not getting full (1/1)*<br>*- Not enough breast-milk (1/1)*<br>*2 months (2/22)*<br>*- Return to work (1/1)*<br>*- Baby was not getting full (1/1)*<br>*3 months (2/22)*<br>*- Low growth/ weight stationary (2/2)*<br>*6 months (9/22)*<br>*9 months (1/22)*<br>*Never fed with formula milk (4/22)*<br><br>***At least 6 months (14/22)***<br>***Before six months (8/22)***<br>*- Not enough breast-milk (3/8)*<br>*- Baby was not getting full (3/8)*<br>*- Low growth/ weight stationary (2/8)*<br>*- Return to work (1/8)*

Setting	FG	Administration of water	Administration of formula milk
	**Summary for all FG**	*At birth/while in maternity (18/54)*<br>- *Breast-milk was not flowing (7/18)*<br>- *Breast-milk is hot (3/18)*<br>- *Grand-mother (2/18)*<br>- *Warmth/very hot (2/18)*<br>- *Baby was thirsty (1/18)*<br>- *Baby was crying a lot (1/18)*<br>- *Others mothers were giving (1/18)*<br>- *Baby was receiving substitute (1/18)*<br>- *My willingness (1/18)*<br>*1 month (2/54)*<br>- *Baby was crying a lot (1/2)*<br>- *People was talking a lot (1/2)*<br>- *Grand-mother (1/2)*<br>*2 months (8/54)*<br>- *Warmth/very hot (4/8)*<br>- *Baby was crying a lot (3/8)*<br>- *People was talking a lot (2/8)*<br>*3 months (5/54)*<br>- *Recommend by health care providers (1/5)*<br>- *Baby was crying a lot (1/5)*<br>- *Baby was disturbing (1/5)*<br>- *Grand-mother (1/5)*<br>- *Warmth/very hot (1/5)*<br>*4 months (3/54)*<br>- *Warmth/very hot (3/3)*<br>*5 months (1/54)*<br>- *Father and hunts (1/1)*<br>*6 months (17/54)*<br><br>***At least 6 months (17/54)***<br>***Before six months (37/54)***<br>- *Warmth/very hot (10/37)*<br>- *Breast-milk was not flowing (7/37)*<br>- *Baby was crying a lot (6/37)*<br>- *Grand-mother (4/37)*<br>- *People was talking a lot (3/37)*<br>- *Breast milk is hot (3/37)*<br>- *Baby was disturbing (1/37)*<br>- *Feather and hunts (1/37)*<br>- *Others mothers were giving (1/37)*<br>- *Baby was thirsty (1/37)*<br>- *Baby was receiving substitute (1/37)*<br>- *Recommend by health care providers (1/37)*<br>- *My willingness (1/37)*	*2 days (1/54)*<br>- *Not enough breast-milk (1/1)*<br>*3 days (1/54)*<br>- *Baby was not getting full (1/1)*<br>- *Not enough breast-milk (1/1)*<br>*1 month (7/54)*<br>- *Low growth/ weight stationary (2/7)*<br>- *Baby was not getting full (1/7)*<br>- *Not enough breast-milk (2/7)*<br>- *Return to work (2/5)*<br>- *For the baby to become accustomed (1/5)*<br>- *Baby was crying a lot (1/5)*<br>- *People was talking (1/5)*<br>- *Recommendations from providers (1/5)*<br>*2 months (5/54)*<br>- *Return to work (2/5)*<br>- *Return to school (1/3)*<br>- *For the baby to become accustomed (1/3)*<br>- *Baby was not getting full (1/1)*<br>- *Baby was suckling a lot (1/3)*<br>*3 months (2/54)*<br>- *Low growth/ weight stationary (2/2)*<br>*4 months (2/54)*<br>- *Supplement breast milk (1/2)*<br>- *Baby was not getting full (1/2)*<br>*5 months (1/54)*<br>- *Baby was crying a lot (1/1)*<br>- *Baby was not getting full (1/1)*<br>*6 months (20/54)*<br>*7 months (1/54)*<br>*8 months (1/54)*<br>*9 months (1/54)*<br>*Never fed with formula milk (12/54)*<br><br>***At least 6 months (35/54)***<br>***Before six months (19/54)***<br>- *Baby was not getting full (5/19)*<br>- *Not enough breast-milk (4/19)*<br>- *Low growth/ weight stationary (4/19)*<br>- *Return to work (4/19)*<br>- *Return to school (1/19)*<br>- *Supplement breast milk (2/19)*<br>- *Baby was crying a lot (2/19)*<br>- *For the baby to become accustomed (2/19)*<br>- *Baby was suckling a lot (1/19)*<br>- *People was talking (1/19)*<br>- *Recommendations from providers (1/19)*

2.3. Administration of porridge and child feeding decision maker

Setting	FG	Administration of porridge	Decision-maker
**KISENSO**	**FG1**	4 months (1/11)<br>- Baby was crying a lot (1/1)<br>5 months (2/11)<br>- Baby was disturbing (1/2)<br>- Nothing (My willingness) (1/2)<br>6 months (8/11)	Mother (7/11)<br>Father (1/11)<br>Mother and father (1/11)<br>Grandmother (2/11)
	**FG2**	3 months (1/7)<br>- Baby was crying a lot (1/1)<br>4 months (1/7)<br>- Baby was crying a lot (1/1)<br>6 months (5/7)	Mother (4/7)<br>Father (3/7)
	**Summary for KISENSO**	*3 months (1/18)*<br>*- Baby was crying a lot (1/1)*<br>*4 months (2/18)*<br>*- Baby was crying a lot (2/2)*<br>*5 months (2/18)*<br>*- Bay was disturbing (1/2)*<br>*- Nothing/My willingness (1/2)*<br>*6 months (13/18)*<br><br>***At least 6 months (13/18)***<br>***Before six months (5/18)***<br>*- Baby was crying a lot (4/5)*<br>*- Baby was disturbing (1/5)*<br>*- Nothing/My willingness (1/5)*	*Mother (11/18)*<br>*Father (4/18)*<br>*Grandmother (2/18)*<br>*Mother and father (1/18)*
**LEMBA**	**FG3**	2 months (1/6)<br>- Refuse formula milk (1/1)<br>3 months (2/6)<br>- They tell me to give (1/2)<br>- Nothing (My willingness) (1/2)<br>5 months (2/6)<br>- Baby was suckling a lot (1/2)<br>- Baby was not getting full (1/2)<br>6 months (1/6)	Mother (1/6)<br>Father (1/6)<br>Grandmother (4/6)
	**FG4**	3 months (2/7)<br>- Baby was not getting full (2/2)<br>4 months (3/7)<br>- Baby was not getting full (1/3)<br>- Baby was crying a lot (1/3)<br>- Baby grew up (1/3)<br>6 months (1/7)<br>Has not yet fed with porridge (1/7)	Mother (5/7)<br>Mother and father (1/7)<br>Grandmother (1/7)
	**Summary for LEMBA**	*2 months (1/13)*<br>*- Refuse formula milk (1/1)*<br>*3 months (4/13)*<br>*- Baby was not getting full (2/4)*<br>*- They tell me to give (1/4)*<br>*- Nothing/My willingness (1/4)*<br>*4 months (3/13)*<br>*- Baby was not getting full (1/3)*<br>*- Baby was crying a lot (1/3)*	*Mother (6/13)*<br>*Grandmother (5/13)*<br>*Mother and father (1/13)*<br>*Father (1/13)*

Setting	FG	Administration of porridge	Decision-maker
		- *Baby grew up (1/3)*<br>*5 months (2/13)*<br>- *Baby was suckling a lot (1/2)*<br>- *Baby was not getting full (1/2)*<br>*6 months (8/13)*<br>*Has not yet fed with porridge (1/13)*<br><br>***At least 6 months (3/13)***<br>***Before six months (10/13)***<br>- *Baby was not getting full (4/13)*<br>- *Baby was crying a lot (1/13)*<br>- *Baby grew up (1/13)*<br>- *Baby was suckling a lot (1/13)*<br>- *Refuse formula milk (1/13)*<br>- *They tell me to give (1/13)*<br>- *Nothing/My willingness (1/13)*	
**NDJILI**	**FG5**	3 months (7/11)<br>- Baby was crying a lot (5/7)<br>- Not enough breast-milk (2/7)<br>- Baby was not getting full (1/7)<br>- Baby was disturbing (1/7)<br>- Baby grew up (1/7)<br>4 months (1/11)<br>- Baby was disturbing (1/1)<br>5 months (1/11)<br>- Baby was crying a lot (1/1)<br>6 months (2/11)	Mother (4/11)<br>Father (2/11)<br>Mother and father (3/11)<br>Grandmother (1/11)<br>Grandfather (1/11)
	**FG6**	3 months (1/11)<br>- weight stationary (1/1)<br>4 months (2/11)<br>- Baby was crying a lot (2/2)<br>- Baby was suckling too strong (1/2)<br>- Baby was not getting full (1/7)<br>5 months (2/11)<br>- Baby was suckling a lot (1/2)<br>- weight stationary (1/2)<br>6 months (6/11)	Mother (6/11)<br>Father (2/11)<br>Grandmother (2/11)<br>Elder sister (1/11)
	**Summary for NDJILI**	*3 months (8/22)*<br>- *Baby was crying a lot (5/8)*<br>- *Not enough breast-milk (2/8)*<br>- *Baby was not getting full (1/8)*<br>- *Baby was disturbing (1/8)*<br>- *Baby grew up (1/8)*<br>- *weight stationary (1/8)*<br>*4 months (3/22)*<br>- *Baby was crying a lot (2/3)*<br>- *Baby was suckling too strong (1/3)*<br>- *Baby was not getting full (1/3)*<br>- *Baby was disturbing (1/3)*<br>*5 months (3/22)*<br>- *Baby was suckling a lot (1/3)*<br>- *weight stationary (1/3)*<br>- *Baby was crying a lot (1/3)*<br>*6 months (8/22)*	*Mother (10/22)*<br>*Father (4/22)*<br>*Grandmother (3/22)*<br>*Mother and father (3/22)*<br>*Grandfather (1/22)*<br>*Elder sister (1/22)*

Setting	FG	Administration of porridge	Decision-maker
		At least 6 months (8/22)<br>***Before six months (14/22)***<br>- *Baby was crying a lot (8/14)*<br>- *Baby was disturbing (2/14)*<br>- *Not enough breast-milk (2/14)*<br>- *Baby was not getting full (2/14)*<br>- *weight stationary (2/14)*<br>- *Baby was suckling too strong (1/3)*<br>- *Baby was suckling a lot (1/3)*<br>- *Baby grew up (1/8)*	
	**Summary for all FG**	*2 months (1/53)*<br>- *Refuse formula milk (1/1)*<br>*3 months (13/53)*<br>- *Baby was crying a lot (6/13)*<br>- *Baby was not getting full (3/13)*<br>- *Not enough breast-milk (2/8)*<br>- *Baby was disturbing (1/8)*<br>- *They tell me to give (1/4)*<br>- *Nothing/My willingness (1/4)*<br>- *Baby grew up (1/8)*<br>- *weight stationary (1/8)*<br>*4 months (8/53)*<br>- *Baby was crying a lot (6/8)*<br>- *Baby was not getting full (2/8)*<br>- *Baby was disturbing (1/8)*<br>- *Baby grew up (1/8)*<br>- *Baby was suckling too strong (1/8)*<br>*5 months (7/53)*<br>- *Baby was suckling a lot (2/7)*<br>- *Baby was not getting full (1/7)*<br>- *Baby was disturbing (1/7)*<br>- *Baby was crying a lot (1/7)*<br>- *Nothing/My willingness (1/7)*<br>- *weight stationary (1/7)*<br>*6 months (23/53)*<br>*Has not yet fed with porridge (1/53)*<br><br>***At least 6 months (24/53)***<br>***Before six months (29/53)***<br>- *Baby was crying a lot (13/29)*<br>- *Baby was not getting full (6/29)*<br>- *Baby was disturbing (3/29)*<br>- *Not enough breast-milk (2/29)*<br>- *Baby grew up (2/29)*<br>- *Nothing/My willingness (2/29)*<br>- *weight stationary (2/29)*<br>- *Baby was suckling a lot (2/29)*<br>- *Baby was suckling too strong (1/29)*<br>- *Refuse formula milk (1/29)*<br>- *They tell me to give (1/29)*	*Mother (27/53)*<br>*Grandmother (10/53)*<br>*Father (9/53)*<br>*Mother and father (5/53)*<br>*Grandfather (1/53)*<br>*Elder sister (1/53)*

3. Perception on the feasibility of the EBF recommendation

Setting	FG	What is your opinion on the EBF recommendation? Why?	Why some mother your community often failed to meet the recommended duration of EBF?
**KISENSO**	**FG1**	Bad recommendation (1/11)<br>Good recommendation (10/11)<br>- Possible/feasible (9/10)<br>- Mistress of the baby (1)<br>- It depends to the baby (1/10)<br>- Impossible/difficult (1/10)<br>Complications when you will want to give him other foods	Have not been interviewed with this question.
	**FG2**	Good recommendation (7/7)<br>- Possible/feasible (7/7)<br>- Mistress of the baby (1)<br>- Many do so (1)<br>- I've already done it (1)	People talk (3/7)<br>Mothers do not eat well (2/7)<br>Mothers are in a hurry (2/7)<br>Baby disturbs (1/7)
	**Summary for KISENSO**	*Bad recommendation (1/18)*<br>*Good recommendation (17/18)*<br>*- Possible/feasible (15/17)*<br>*- Mistress of the baby (2)*<br>*- Many do so (1)*<br>*- I've already done it (1)*<br>*- It depends to the baby (1/17)*<br>*- Impossible/difficult (1/17)*<br>*Complications when you will want to give him other foods*	*People talk (3/7)*<br>*Mothers do not eat well (2/7)*<br>*Mothers are in a hurry (2/7)*<br>*Baby disturbs (1/7)*
**LEMBA**	**FG3**	Good recommendation (6/6)<br>- Possible/feasible (1/6)<br>- The mother has to be patient and available<br>- Impossible/difficult (5/6)<br>- I've never respect (1)<br>- Babies cry of hungry (3)	Baby cries a lot /disturbs (4/6)<br>Baby does not sleep over night (3/6)<br>People talk a lot (1/6)<br>Health care providers do the same (1/6)
	**FG4**	Bad recommendation (2/7)<br>Good recommendation (5/7)<br>- Possible/feasible (1/5)<br>- Impossible/difficult (4/5)<br>- Babies cry/disturb (1)<br>- The warm/hot climate (1)<br>- Punishes/hurts the baby (3)	Baby cries a lot /disturbs (3/7)<br>Baby does not sleep over night (2/7)<br>People talk a lot (Saying of others) (1/7)<br>Mother's work (1/7)<br>Mothers do not eat well (3/7)
	**Summary for LEMBA**	*Bad recommendation (2/13)*<br>*Good recommendation (11/13)*<br>*- Possible/feasible (2/11)*<br>*- The mother has to be patient and available*<br>*- Impossible/difficult (9/11)*<br>*- I've never respect (1)*<br>*- Babies cry of hungry (3)*<br>*- Babies cry/disturb (1)*<br>*- The warm/hot climate (1)*<br>*- Punishes/hurts the baby (3)*	*Baby cries a lot /disturbs (7/13)*<br>*Baby does not sleep over night (5/13)*<br>*People talk a lot (Saying of others) (2/13)*<br>*Health care providers do the same (1/13)*<br>*Mother's work (1/13)*<br>*Mothers do not eat well (3/13)*

Setting	FG	What is your opinion on the EBF recommendation? Why?	Why some mother your community often failed to meet the recommended duration of EBF?
**NDJILI**	**FG5**	Good recommendation (11/11)<br>- Possible/feasible (7/11)<br>- Mother should not listen to what others say (2)<br>- Mother must resist/persevere (2)<br>- Many do so (1)<br><br>- Impossible/difficult (4/11)<br>- Mothers do not eat well (3)<br>- Babies cry of hunger (1)	Baby cries a lot /disturbs (3/11)<br>Baby does not sleep over night (1/11)<br>Baby stares at those who eat (1/11)<br>Baby don't gain weight (1/11)<br>Baby does not get full (4/11)<br>People talk a lot (1/11)
	**FG6**	Good recommendation (11/11)<br>- Possible/feasible (11/11)<br>- Many do so (1)<br>- Mother must be available (1)	Warm/hot climate (4/7)<br>Saying of others (2/7)
	**Summary for NDJILI**	*Good recommendation (22/22)*<br>*- Possible/feasible (18/22)*<br>*- Mother should not listen to what others say (2)*<br>*- Mother must resist/persevere (2)*<br>*- Many do so (2)*<br>*- Mother must be available (1)*<br><br>*- Impossible/difficult (4/22)*<br>*- Mothers do not eat well (3)*<br>*- Babies cry of hunger (1)*	*Warm/hot climate (4/18)*<br>*Baby does not get full (4/18)*<br>*Baby cries a lot /disturbs (3/18)*<br>*People talk a lot/Saying of others (3/18)*<br>*Baby does not sleep over night (1/18)*<br>*Baby stares at those who eat (1/18)*<br>*Baby don't gain weight (1/18)*
	**Summary for all FG**	*Bad recommendation (3/53)*<br>*Good recommendation (50/53)*<br>*- Possible/feasible (34/50)*<br>*- The mother has to be patient and available (2)*<br>*- Mistress of the baby (2)*<br>*- Many do so (3)*<br>*- I've already done it (1)*<br>*- Mother should not listen to what others say (2)*<br>*- Mother must resist/persevere (2)*<br><br>*- It depends to the baby (1/50)*<br><br>*- Impossible/difficult (14/50)*<br>*- I've never respect (1)*<br>*- Babies cry of hungry (4)*<br>*- Babies cry/disturb (1)*<br>*- The warm/hot climate (1)*<br>*- Punishes/hurts the baby (3)*<br>*- Mothers do not eat well (3)*<br>*- Complications when you will want to give him other foods*	*Baby cries a lot /disturbs (11/38)*<br>*People talk a lot/Saying of others (8/38)*<br>*Baby does not sleep over night (6/38)*<br>*Mothers do not eat well (5/38)*<br>*Warm/hot climate (4/38)*<br>*Baby does not get full (4/38)*<br>*Mothers are in a hurry (2/38)*<br>*Baby stares at those who eat (1/38)*<br>*Baby don't gain weight (1/38)*<br>*Mother's work (1/38)*<br>*Health care providers do the same (1/38)*

4. Perceived social norm regarding breastfeeding and EBF

Setting	FG	Social norm about EBF/What is the opinion of people in your community on the practice of EBF?	Breastfeeding in public transportation/Bus
**KISENSO**	**FG1**	They do not agree (9/10)<br>- People talk a lot<br>- Boastful (2)<br>- starve/hurt the child (2)<br>- Elderly mothers (1)<br>- We used to give water, … what wrong happened?<br>Some agree (1/10)<br><br>The mother has to<br>- Be firm (1)<br>- Decide/decision (1)	Do breastfeed (11/12)<br>Do not breastfeed (1/12)
	**FG2**	They do not agree (6/6)<br>- People talk a lot<br>- Boastful (2)<br>- Hurt the child (1)<br>- Elderly mothers (2)<br>- We used to give water, … what wrong happened?<br>Some agree (1/6)<br>- Encourage mothers<br><br>The mother has to<br>- Decide/decision (1)	Do breastfeed (7/7)<br><br>People are ok with breastfeeding on the bus (7/7)<br>- If the baby is crying, they will ask you breastfeed him
	**Summary for KISENSO**	*They do not agree (15/16)*<br>- *People talk a lot (7)*<br>- *Boastful (4)*<br>- *starve/hurt the child (3)*<br>- *Elderly mothers (3)*<br>- *We used to give water, … what wrong happened? (3)*<br>*Some agree (2/16)*<br>- *Encourage mothers (1)*<br><br>*The mother has to*<br>- *Be firm (1)*<br>- *Decide/decision (2)*	*Do breastfeed (18/19)*<br>*Do not breastfeed (1/19)*<br><br>*People are ok with breastfeeding on the bus (7/7)*<br>- *If the baby is crying, they will ask you breastfeed him*
**LEMBA**	**FG3**	They do not agree (6/6)<br>- People talk a lot<br>- It won't do anything (4)<br>- Elderly mothers (1<br>- We used to give water, … what wrong happened?	Do breastfeed (4/6)<br>Do not breastfeed (2/6)<br><br>People are ok with breastfeeding on the bus (4/6)<br>- If the baby is crying, they will ask you breastfeed him
	**FG4**	They do not agree (4/7)<br>- People talk a lot<br>- Health care providers do not respect (1)<br>Some agree (2/7)<br>The mother has to<br>- Decide/decision (2)	Do breastfeed (4/6)<br>Do not breastfeed (2/6)

Setting	FG	Social norm about EBF/What is the opinion of people in your community on the practice of EBF?	Breastfeeding in public transportation/Bus
	**Summary for LEMBA**	*They do not agree (10/13)*<br>- *People talk a lot (5)*<br>- *Health care providers do not respect (1)*<br>- *It won't do anything (4)*<br>- *Elderly mothers (1)*<br>- *We used to give water, … what wrong happened? (1)*<br>*Some agree (2/13)*<br><br>*The mother has to*<br>- *Decide/decision (2)*	*Do breastfeed (8/12)*<br>*Do not breastfeed (4/12)*<br><br>*People are ok with breastfeeding on the bus (4/6)*<br>- *If the baby is crying, they will ask you breastfeed him*
**NDJILI**	**FG5**	They do not agree (10/11)<br>- People talk a lot<br>- Starve the child (1)<br>- Killing the child (2)<br>- Elderly mothers (3)<br>- We used to give water, … what wrong happened?<br>Some agree (1/11)<br><br>The mother has to<br>- Resist (1)	Do breastfeed (9/11)<br>- Cover the breast (3/9)<br>Do not breastfeed (2/9)<br><br>People are ok with breastfeeding on the bus (9/11)<br>- If the baby is crying, they will ask you breastfeed him
	**FG6**	They do not agree (7/7)<br>- People talk a lot<br>- Starve the child (1)<br>- Elderly mothers (1)<br>- We used to give water, … what wrong happened?<br>The mother has to<br>- Resist (1)	Do breastfeed (7/7)<br><br>People are ok with breastfeeding on the bus (7/7)<br>- If the baby is crying, they will ask you breastfeed him
	**Summary for NDJILI**	*They do not agree (17/18)*<br>- *People talk a lot (4)*<br>- *Starve the child (2)*<br>- *Killing the child (2)*<br>- *Elderly mothers (3)*<br>- *We used to give water, … what wrong happened? (3)*<br>*Some agree (1/18)*<br>*The mother has to*<br>- *Resist (2)*	*Do breastfeed (16/18)*<br>- *Cover the breast (3/16)*<br>*Do not breastfeed (2/18)*<br><br>*People are ok with breastfeeding on the bus (16/17)*<br>- *If the baby is crying, they will ask you breastfeed him*
	**Summary for all FG**	*They do not agree (42/47)*<br>- *People talk a lot (16)*<br>- *Boastful (4)*<br>- *starve/hurt the child (5)*<br>- *Killing the child (2)*<br>- *Health care providers do not respect (1)*<br>- *It won't do anything (4)*<br>- *Elderly mothers (7)*<br>- *We used to give water, … what wrong happened? (7)*	*Do breastfeed (42/49)*<br>- *Cover the breast (3/42)*<br>*Do not breastfeed (7/49)*<br><br>*People are ok with breastfeeding on the bus (27/30)*<br>- *If the baby is crying, they will ask you breastfeed him*

Setting	FG	Social norm about EBF/What is the opinion of people in your community on the practice of EBF?	Breastfeeding in public transportation/Bus
		Some agree (5/47)<br>- *Encourage mothers (1)*<br><br>*The mother has to*<br>- *Decide/decision (4)*<br>- *Resist (2)*<br>- *Be firm (1)*	
