# Supplementary material for: Breastfeeding practices and social norms in Kinshasa, Democratic Republic of the Congo: A qualitative study
Source: PLOS Glob Public Health. 2024 Apr 16;4(4):e0000957. doi: 10.1371/journal.pgph.0000957 (PMC11020689; doi:10.1371/journal.pgph.0000957)
Supplement: S2 File — (DOCX) [file pgph.0000957.s002.docx]

**FOCUS GROUP MODERATOR'S GUIDE**

**Introduction**

Welcome and thank you for taking the time to participate in this discussion. My name is Pélagie BABAKAZO, I'm a doctor and I'm interested in children's nutrition. And this one is Madame Nelly Lobota, who will help us to take note.

We want to talk to you about feeding children under six months. We want to understand what these children eat and why they eat it. The answers you will give us during this group discussion will enable to propose effective interventions to improve the diet of children of this age, and consequently their health.

# Rules of Conduct

# During this discussion, each of your opinions is important and of great interest to us. Everyone's point of view counts. So, we encourage you to speak up. There are no right or wrong answers. No one will be criticized for what you will say. When you speak, we ask you to be sincere so that we can obtain quality information.

# Some of you may or may not agree with other people's answers, which is normal. However, we urge you not to interrupt others while they are speaking. The floor will be given to each of you in turn, and everyone will have time to speak. Please respect each other's point of view, even if it differs from your own. You may refuse to answer any question that makes you feel uncomfortable.

# Our group discussion will last an hour and a half on average.

# Confidentiality and use of Dictaphone

# Everything said here must be kept confidential. No one should talk about what is said here to other people. Furthermore, we will not need your names, we will use the numbers to identify each of you during the discussion. My assistant will take note of your answers and a Dictaphone will be used to record everything that will be said and therefore allow us to correct the notes taken. Do you agree that we can record this discussion?

# So, can we start?

1. **Breastfeeding practices in the first six months**

Q1. How have you fed your baby during the two first days after birth?

Q2. How old was your baby when you started giving him/her water; formula milk; porridge? Why did you do so at that moment?

1. **Knowledge of breastfeeding**

Q3. What are the advantages of breastfeeding?

Q4. What are the disadvantages of breast-feeding?

Q5. To your knowledge, how long should a child be exclusively breastfed?

1. **Perception of the feasibility of EBF recommendation**

Q6. What do you think about the feasibility of the recommendation that mothers should only breastfeed their babies for the first six months?

Q7. Why do some mothers in your community often fail to meet the recommended duration of EBF?

1. **Perceived social norms regarding breastfeeding and exclusive breastfeeding**

Q8. What do you think about breastfeeding in public?

Q9. What do you think of the perceptions and attitudes of people in Kinshasa towards breastfeeding and EBF?

Thank you for taking the time to participate in this discussion. We also thank you for your active participation.
